# Single-cell RNA-seq analysis identifies distinct myeloid cells in a case with encephalitis temporally associated with COVID-19 vaccination

**DOI:** 10.3389/fimmu.2023.998233

**Published:** 2023-02-23

**Authors:** Masakazu Ishikawa, Yuki Shimada, Tatsuhiko Ozono, Hisatake Matsumoto, Hiroshi Ogura, Keigo Kihara, Hideki Mochizuki, Tatsusada Okuno, Shuhei Sakakibara, Makoto Kinoshita, Daisuke Okuzaki

**Affiliations:** ^1^ Laboratory of Human Immunology (Single Cell Genomics), WPI Immunology Frontier Research Center, Osaka University, Osaka, Japan; ^2^ Center for Infectious Disease Education and Research, Osaka University, Osaka, Japan; ^3^ Department of Neurology, Graduate School of Medicine, Osaka University, Osaka, Japan; ^4^ Department of Traumatology and Acute Critical Medicine, Graduate School of Medicine, Osaka University, Osaka, Japan; ^5^ Laboratory of Immune Regulation, WPI Immunology Frontier Research Center, Osaka University, Osaka, Japan; ^6^ Genome Information Research Center, Research Institute for Microbial Diseases, Osaka University, Osaka, Japan; ^7^ Institute for Open and Transdisciplinary Research Initiatives, Osaka University, Osaka, Japan

**Keywords:** single-cell RNA-seq, myeloid cells, COVID-19, vaccination, encephalitis

## Abstract

Recently accumulating evidence has highlighted the rare occurrence of COVID-19 vaccination-induced inflammation in the central nervous system. However, the precise information on immune dysregulation related to the COVID-19 vaccination-associated autoimmunity remains elusive. Here we report a case of encephalitis temporally associated with COVID-19 vaccination, where single-cell RNA sequencing (scRNA-seq) analysis was applied to elucidate the distinct immune signature in the peripheral immune system. Peripheral blood mononuclear cells (PBMCs) were analyzed using scRNA-seq to clarify the cellular components of the patients in the acute and remission phases of the disease. The data obtained were compared to those acquired from a healthy cohort. The scRNA-seq analysis identified a distinct myeloid cell population in PBMCs during the acute phase of encephalitis. This specific myeloid population was detected neither in the remission phase of the disease nor in the healthy cohort. Our findings illustrate induction of a unique myeloid subset in encephalitis temporally associated with COVID-19 vaccination. Further research into the dysregulated immune signature of COVID-19 vaccination-associated autoimmunity including the cerebrospinal fluid (CSF) cells of central nervous system (CNS) is warranted to clarify the pathogenic role of the myeloid subset observed in our study.

## Introduction

The outbreak of severe acute respiratory syndrome called coronavirus disease-2019 (COVID-19) was caused by a novel coronavirus (SARS-CoV-2) infection (1). The SARS-CoV-2 infection has spread rapidly worldwide by high human-to-human transmission, resulting in a public health emergency of international concern. The ongoing COVID-19 pandemic has been described as ‘an explosive pandemic of historic proportions’, with over 200 million confirmed cases and over 5 million confirmed deaths worldwide (2). Several mRNA vaccination applications have prevented severe SARS-CoV-2 disease outcomes (3). Accumulating evidence demonstrates that mRNA vaccination is highly effective in eliciting the production of antibodies against SARS-CoV-2 (3–5). Despite the well-acknowledged efficacy of mRNA vaccination of SARS-CoV-2, the precise alteration of immune responses elicited by mRNA vaccination remains to be clarified. Although several studies suggest the safety of mRNA vaccination for patients suffering from autoimmune neurological diseases (6, 7), reports showing the rare occurrence of autoimmune diseases affecting peripheral or central nervous system is accumulating (8–10). The frequency of encephalitis after COVID-19 mRNA vaccination is estimated to be 2 in 10 million (11). Thus, it is crucial to clarify the immune dysregulation triggered and identify the cellular population contributing to the development of COVID-19 vaccination associated-autoimmunity. Improvements in DNA library preparation technology for sequencing have enabled RNA sequencing to comprehensively analyze gene expression levels at the single-cell level (12). Single cell RNA-sequencing (scRNA-seq) provides information on both the proportion of each specific cellular subset, and the gene signature of the cells (13). Here, we describe a case where augmentation of autoimmune encephalitis was observed after COVID-19 vaccination using scRNA-seq of peripheral blood mononuclear cells (PBMCs), and further demonstrate distinct myeloid cell population identified in PBMCs at the acute phase of the disease.

## Materials and methods

### Subjects and PBMC preparation

PBMCs were chronologically collected from the patient at the onset of encephalitis (day 0), two days after the onset of the disease (day 3), and at the remission of the disease (day 17). Written informed consent was obtained from the participant prior to the participation in the study. The protocol was reviewed and approved by the Ethics Committee of Osaka University and in accordance with the tenets set forth in the Declaration of Helsinki.

### Isolation of PBMCs

PBMCs were isolated using Histopaque 1077 (Sigma) by centrifugation at 800g for 15 min at room temperature. PBMCs at the interface were collected, rinsed twice with phosphate buffered saline (PBS) and 2% bovine serum albumin (BSA), and cryopreserved in fetal bovine serum with 10% dimethyl sulfoxide. All samples were processed within 4 hours of collection. The preserved PBMCs were thawed immediately at 37°C, transferred to a 50-ml tube, and ten volumes of prewarmed PBS was added slowly and dropwisely, followed by centrifugation at 500g for 5 min. The pellet was resuspended in 1 ml of PBS with 2% BSA, and the viability of each sample was assessed by counting using trypan blue and a Countess II FL Automated Cell Counter (Thermo Fisher Scientific).

### TotalSeq-C hashtag antibody staining, single cell library preparation and sequencing

The PBMCs from each donor were stained with Human TruStain FcX Fc Blocking Reagent (BioLegend, 422302) for 10 min at 4°C. Subsequently, the cells were then stained with a TotalSeq-C hashtag (BioLegend) for 30 min at 4°C. The cells were then washed twice using centrifugation at 500g for 5 min at 4°C with PBS supplemented with 2% (vol/vol) BSA. Each sample’s cell number and viability were determined using trypan blue and a Countess II FL Automated Cell Counter, then pooled together in equal numbers. The cells were counted again and processed immediately for a 10x 5’ single-cell system followed by Chromium Next GEM Single Cell V(D)J Reagent Kit v2 with Feature Barcoding technology for Cell-Surface Protein-Rev D protocol. Gene expression and feature barcode libraries were prepared according to the manufacturer’s protocol (10x Genomics). All libraries were sequenced using the DNBSEQ-G400 (MGI) to achieve a minimum of 20,000 paired-end reads per cell for gene expression and 5,000 paired-end reads per cell for cell-surface protein.

### Bioinformatics analysis

Sequencing data obtained from MGISEQ-G400 were aligned to the GRCh38 genome using Cell Ranger (v.6.1.0). We have also obtained the public data from Wang et al. (2022) (reference number: OMIX001295) as healthy subjects, which samples were analyzed immediately after the second dose of mRNA-1273 vaccination (14). Filtered matrices were loaded into the R package Seurat (v.4.0) (15) and conducted data filtering, normalization, scaling, dimensional reduction, clustering, and visualization were conducted using Seurat. After clustering, cell types were automatically determined by using ScType (16). The gene expression information of surface proteins is shown in **Supplementary Table 1**. Differential gene expression (DE) analysis was conducted by FindMarker script implemented in Seurat. The results of DE analysis were used in volcano plot and Gene Ontology (GO) enrichment analysis. The figure of the volcano plot was plotted by hand-made scripts, and GO enrichment analysis was conducted by using the compareCluster function in the R package clusterProfiler (17). Genes differentially expressed were identified as p_val_adj < 0.1 and |Log2 FC| > 1.

### Data availability

Data that support the findings of this study are available from the corresponding author upon reasonable request. Data for all scRNA-seq will be available through GEO at accession number GSE205606 and GSE205607.

## Results

### Case presentation

**Figure 1:**
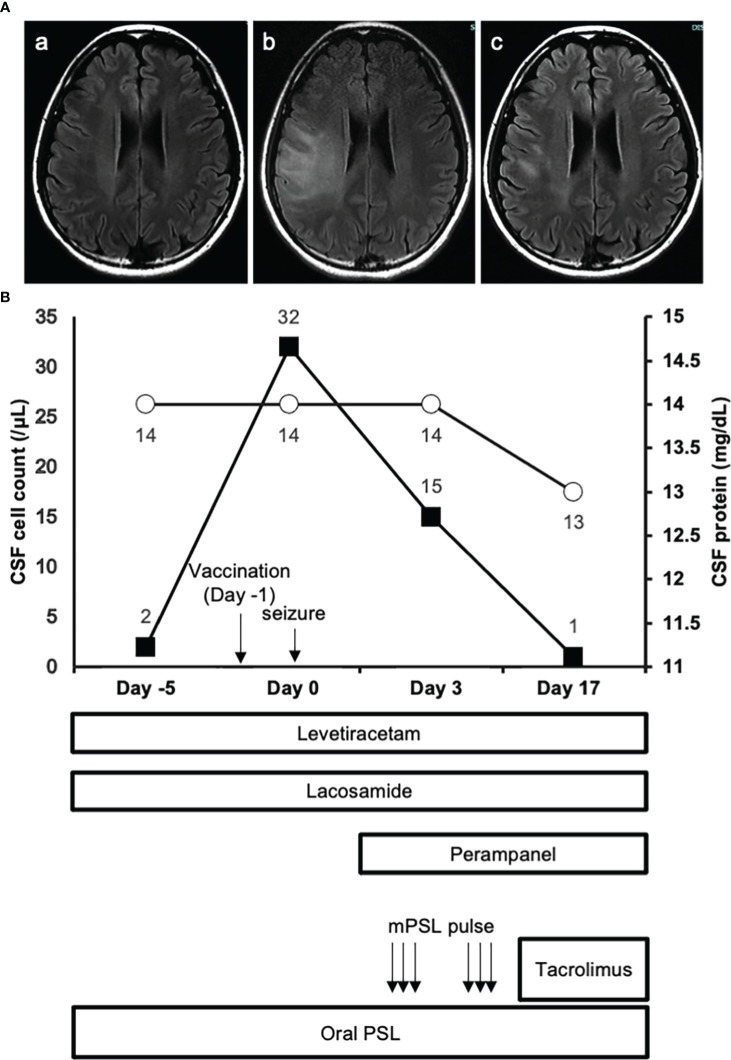
MRI images and clinical course. **(A.a)** FLAIR image of the brain obtained 2 days before the vaccination. **(A.b)** FLAIR image of the brain on day 0 show extensive development of high intensity lesions predominantly at the white matter. **(A.c)** FLAIR image of the brain on day 24 reveals the amelioration of high intensity lesions observed at the acute phase of the disease. **(B)** The closed squares and open circles represent CSF cell count and protein concentration of each timepoint respectively.

A 25-year-old Asian woman had been experiencing swelling and pain in her right toe interphalangeal joint and finger proximal interphalangeal joint. She was diagnosed with rheumatoid arthritis and commenced treatment with methotrexate after a positive blood test for rheumatoid factor and joint echo results. Subsequently, she developed a generalized convulsive seizure and was transferred to her previous physician. MRI T2-weighted head images showed high-signal areas just below the cerebral cortex in the right frontal and parietal lobes, and both symptoms and imaging findings improved with antiepileptic drugs and oral steroids. Accordingly, the patient was discharged with a diagnosis of autoimmune encephalitis associated with the extra-articular manifestation of rheumatoid arthritis. Four months later, the seizures recurred again, and the patient was transferred to our hospital for specialist care with increased doses of oral steroids and antiepileptic drugs. At the time of transfer, there were no obvious neurological abnormalities, and CSF examination was normal. The patient was scheduled for discharge from the hospital after a gradual reduction of steroids. However, when the second dose of COVID-19 vaccination (mRNA-1273) was administered in the same month, the patient developed fever during the night on the same day and generalized tonic-clonic seizures in the early morning of the next day (day 0). MRI images of the head revealed a high-signal area in the subcortical white matter in the fluid-attenuated inversion recovery scan (**Figure 1A**), and CSF examination showed an elevated cell count of 32 cells/μL (**Figure 1B**). The patient was ventilated for seizures and treated with diazepam, fosphenytoin, midazolam, and propofol. Three days later (day 3), the patient was extubated and treated with steroid pulse therapy, and tacrolimus was introduced in addition to oral steroids to prevent further relapses of encephalitis. No residual neurological symptoms were observed, and the patient was discharged from the hospital. On day 17 spinal fluid findings were normalized, and the lesion had markedly resolved on head MRI imaging (**Figures 1A, B**).

### Distinct myeloid cell population can be observed in the acute phase of encephalitis

**Figure 2:**
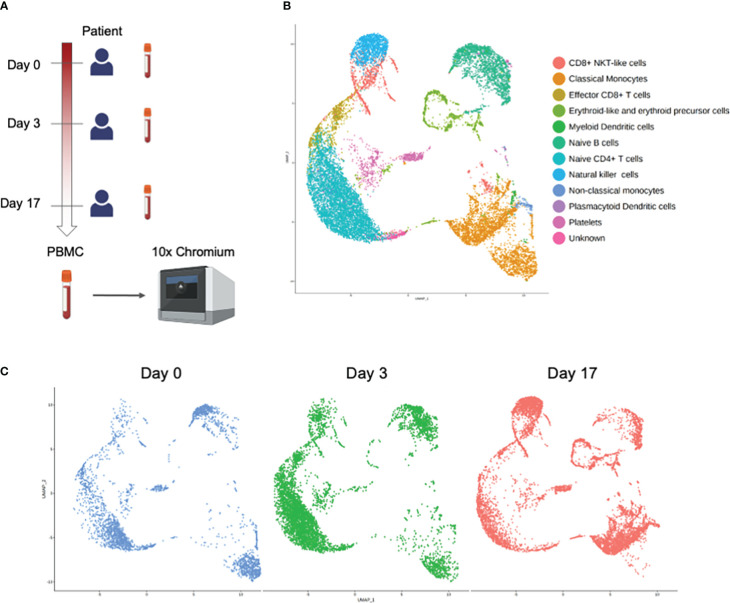
Single-cell RNA-seq analysis of PBMCs obtained at day 0, day 3, and day 17 from the patient. **(A)** Experimental design of the study. Figure was created using BioRender.com. **(B)** UMAP projection of all PBMCs with major subsets annotated. **(C)** UMAP projection of all PBMCs split by samples.

We generated single-cell transcriptome data of PBMCs obtained from the patient at day 0, day 3 and day 17 (**Figure 2A**). We obtained 16,295 cells in total from the 3 conditions after doublet removal. **Figure 2B** shows the landscape of each immune subset after analyzing the combined scRNA-seq results of the 3 samples. When scRNA-seq results of each sample were analyzed respectively, PBMCs obtained at day 0 and day 3 of the patient revealed the appearance of a distinct cellular population compared to those of day 17 in the cluster island annotated as classical monocytes (**Figure 2C**). There were no other distinct cellular subsets or clonal predominance observed in the acute phase of the disease (**Supplementary Tables 2, 3**).

### Immunological pathways specific to the distinct myeloid cell population at the acute phase of encephalitis

**Figure 3:**
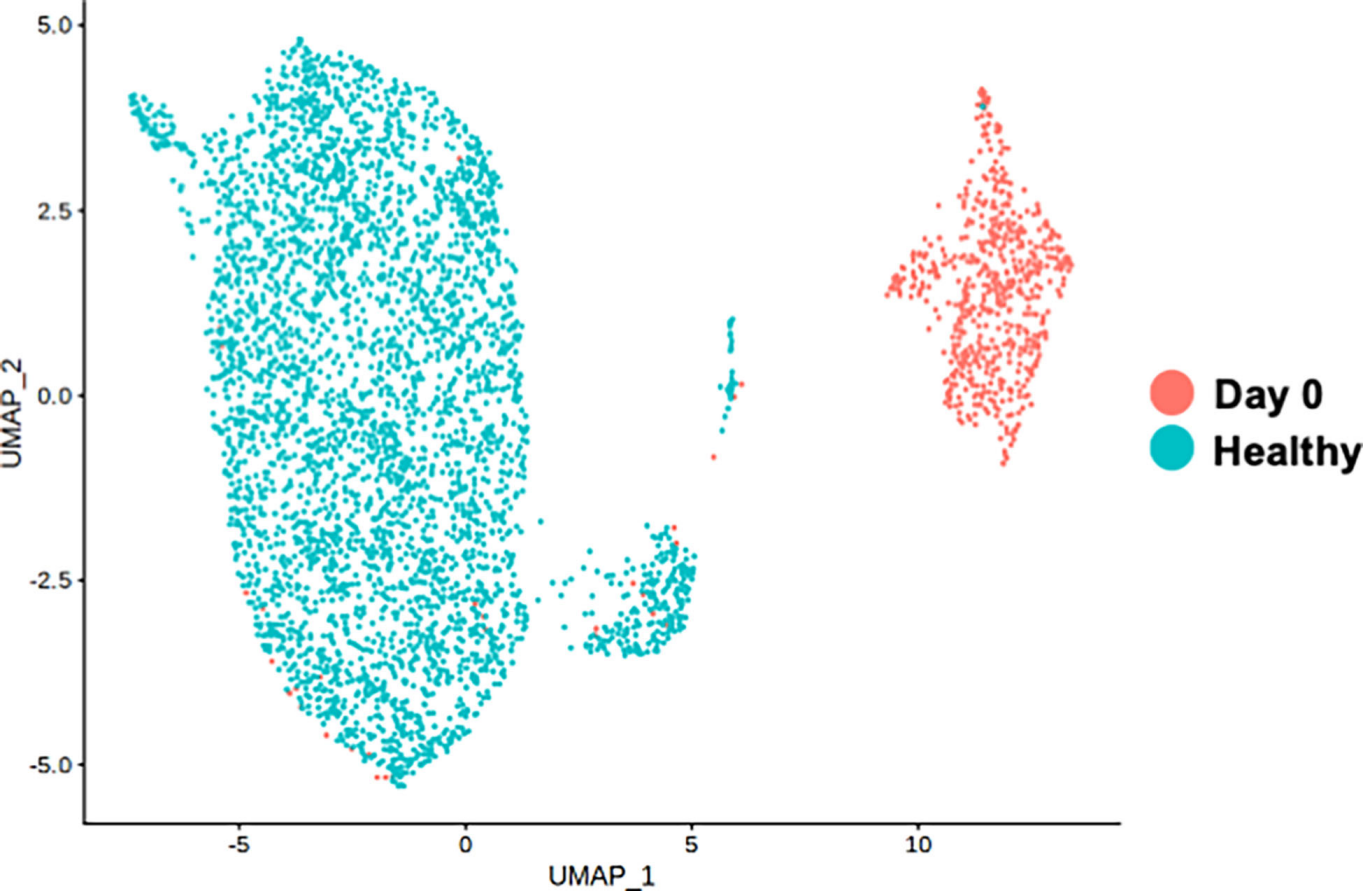
Single-cell RNA-seq analysis of classical monocyte cell population. UMAP visualization of classical monocytes colored by sample conditions. “Day 0” depicts the samples obtained from the patient on day 0. “Healthy” depicts the samples obtained from the public healthy control data OMIX001295.

To clarify whether the distinctive classical monocytes observed at the acute phase of the disease in our patient was not the immune alteration shared with healthy subjects receiving vaccination, PBMCs obtained at day 0 were compared with the healthy controls receiving COVID-19 vaccination from public database. Both samples were obtained one day after the mRNA-1273 vaccine. **Figure 3** shows the distinct monocyte clusters between day 0 of the patient and the healthy controls. Differential expression gene (DEG) analysis was further performed among the classical monocytes between day 0 of the patient and the healthy controls. DEGs were defined by the threshold as p_val_adj < 0.1 and |Log2Fold| > 1. The most highly up-regulated genes of the classical monocytes of day 0 were *G0S2* (G0/G1 Switch 2), *TIMP1* (TIMP Metallopeptidase Inhibitor 1), *ASPH* (Aspartate Beta-Hydroxylase), and *HMOX1* (Heme Oxygenase 1), whereas the most prominently down-regulated genes were *FOS* (Fos Proto-Oncogene), *DUSP1* (Dual Specificity Phosphatase 1), *RHOB* (Ras Homolog Family Member B), and *MNDA* (Myeloid Cell Nuclear Differentiation Antigen) (**Supplementary Table 4**).

**Figure 4:**
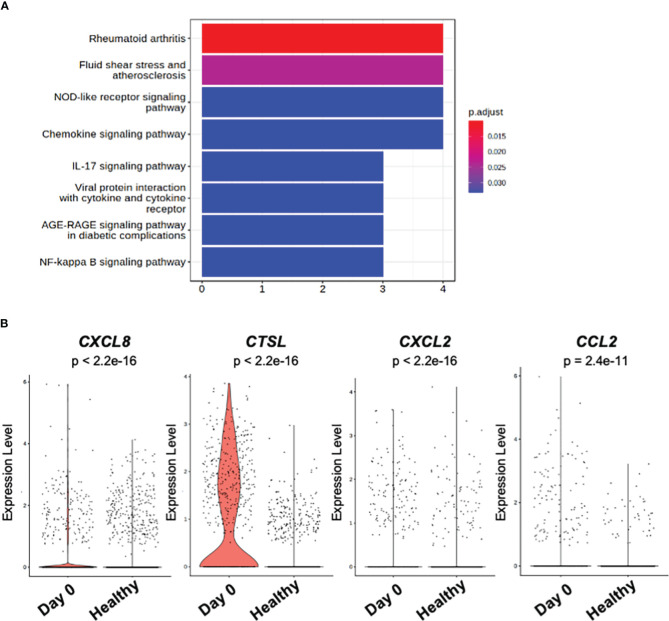
KEGG pathway and DEG analysis of classical monocytes. **(A)** Bar plot of KEGG pathway enriched in significantly (p_val_adj < 0.1 and |avg_log2FC| > 1) up-regulated genes among classical monocytes at day 0 and day 3 of the patient. **(B)** Violin plot of the genes related to KEGG pathway term “Rheumatoid arthritis” in **(A)**. “Day 0” depicts the samples obtained from the patient on day 0. “Healthy” depicts the samples obtained from the public healthy control data OMIX001295.

To further elucidate the molecular pathway representing the overall gene signature characteristic to the distinct monocyte cluster observed at the acute phase of day 0, Kyoto Encyclopedia of Genes and Genomes Enrichment analysis (KEGG) Enrichment analysis was performed. As shown in **Figure 4A**, the pathway term “Rheumatoid arthritis” represented the up-regulated gene signature of the monocyte cluster observed at day 0. No pathway terms were enriched for genes down-regulated in the monocyte cluster.

The DEGs which contributed to the pathway term “Rheumatoid arthritis” were *CXCL8* (C-X-C Motif Chemokine Ligand 8), *CTSL* (Cathepsin L), *CXCL2* (C-X-C Motif Chemokine Ligand 2), and *CCL2* (C-C Motif Chemokine Ligand 2) (**Figure 4B**).

## Discussion

In the COVID-19 pandemic era, clinical trials have revealed that mRNA vaccines, a novel vaccine modality, prevent COVID-19 infection at a high rate and reduce the risk of severe disease (3). Adverse reactions are not severe in the majority of vaccine recipients; however, rare adverse reactions of autoimmune neurological diseases have been reported (8–10).

Typical COVID-19 vaccination-related autoimmune neurological diseases reported include cranial nerve palsies (18), Guillain-Barré syndrome (9, 19), myelitis (20), and encephalitis (10), but the details of the altered immune responses that contribute to their pathogenesis remain unresolved.

Our patient is a rare case of rheumatoid encephalitis with acute exacerbation, which was observed after the vaccine booster immunization. It remains elusive whether the specific classical monocyte population identified in our case is observed in COVID-19 vaccination-related CNS diseases in general, or is rather reflecting enhanced dysregulated immunity of each specific disease. Accumulating evidence is warranted to clarify the potential role of the specific classical monocyte population to utilize as the surrogate marker of immune flare in COVID-19 vaccination-related CNS diseases.

Recent reports of detailed single-cell analysis after mRNA vaccine administration have revealed that vaccination induces specific acquired immune activation, including antigen-specific CD4-positive T cells and CD8-positive T cells, while booster vaccination induces notably enhanced innate immune responses (21). The enhanced responses of CD4- and CD8-positive T cells after the booster vaccination is also demonstrated in another study (22), while memory B cells are also demonstrated to be primed by mRNA vaccine (23). Furthermore, immunosuppressant medication is shown to inhibit the efficacious germinal center responses elicited by mRNA vaccine (24).

The specific classical monocyte population we identified in this study is characterized by high expression of CXCL8, CTSL, CXCL2, and CCL2, all of which are molecules associated with rheumatic disease activity, and we hypothesized that the COVID-19 vaccination may have been the trigger for the rheumatic activity in our patients. CXCL8 is known to be elevated in PBMCs of patients with active rheumatoid arthritis (25). The cathepsins including CTSL is known to be expressed at high levels in the joints of rheumatoid arthritis (26). It is also reported that CXCL2 is significantly elevated in the serum of rheumatoid arthritis compared to healthy controls (27). In addition, CCL2 has been reported to be elevated in the serum of rheumatoid patients compared to healthy controls (28).

Large clinical studies of rheumatoid arthritis have reported that COVID-19 vaccine does not clearly increase the risk of recurrence (29), but there are rare reports of increased disease activity and recurrence (30). Rheumatoid arthritis is mainly characterized by joint symptoms, but can be complicated by various central nervous system symptoms, including meningitis and encephalitis (31).

It remains to be clarified whether the specific classical monocyte population we identified in this study directly contributes to the development of encephalitis by infiltrating the brain or by enhancing systemic production of inflammatory cytokines, leading to brain lesions. In this regard, further analysis of CSF cells, which was not feasible in our study due to insufficient number of cells collected, will provide more convincing evidence to show the pathogenic immune subsets responsible for encephalitis development after COVID-19 vaccination.

Another limitation of this study is that the patients were under treatment with various types of medication at the time of sample collection. In this regard, we cannot exclude the possibility that these multi-factorial effects altered the gene expression pattern of our scRNA-seq results of the patient.

Considering the autoimmune background of rheumatoid arthritis in our patient, it is also interesting to elucidate whether COVID-19 vaccination can activate the immune signature underlying the pathogenesis characteristic of each disease related to autoimmunity in the future studies.

## Data availability statement

Data that supports the findings of this study are available from the corresponding author upon reasonable request. Data for all scRNA-seq are available through GEO at accession number GSE205606 and GSE205607.

## Ethics statement

The studies involving human participants were reviewed and approved by the Ethics Committee of Osaka University. The patients/participants provided their written informed consent to participate in this study. Written informed consent was obtained from the individual(s) for the publication of any potentially identifiable images or data included in this article.

## Author contributions

MI, SS, MK, and DO designed research; MI, YS, TOz, HMa, HO, KK, HMo, TOk, SS, MK, and DO performed research; MI, SS, MK, and DO analyzed data; MI, SY, and MK wrote the paper. All authors contributed to the article and approved the submitted version.
